# miR-494 Contributes to Estrogen Protection of Cardiomyocytes Against Oxidative Stress *via* Targeting (NF-κB) Repressing Factor

**DOI:** 10.3389/fendo.2018.00215

**Published:** 2018-05-14

**Authors:** Zhi-Ping Tang, Wei Zhao, Jian-kui Du, Xin Ni, Xiao-Yan Zhu, Jian-Qiang Lu

**Affiliations:** ^1^The Key Laboratory of Exercise and Health Sciences of Ministry of Education, School of Kinesiology, Shanghai University of Sport, Shanghai, China; ^2^Department of Physiology, Second Military Medical University, Shanghai, China; ^3^Research Laboratory of Burn and Trauma, PLA 181 Hospital, Guilin, China

**Keywords:** miR-494, estrogen, cardiomyocytes, oxidative stress, (NF-κB) repressing factor

## Abstract

Oxidative stress plays a pivotal role in the initiation and progression of cardiac diseases. Estrogens have been demonstrated to exert pleiotropic cardioprotective effects, among which antioxidative stress is one of the key effects linking estrogens to cardioprotection. By using a microRNAs (miRs) microarray screening approach, we discovered an increase in miR-494, which is known to exert cardioprotective effects, in estrogen-treated cardiomyocytes. We hypothesized that the upregulation of miR-494 might contribute to estrogen-mediated cardioprotection against oxidative stress. We found that E_2_ stimulates miR-494 expression *via* ERα in both cardiomyocytes and the myocardium of female mice. The miR-494 inhibitor attenuated the protective effect of 17β-estradiol (E_2_) against oxidative stress-induced injury in cardiomyocytes. By contrast, the miR-494 mimic protected cardiomyocytes against oxidative stress-induced cardiomyocyte injury. Using real-time PCR, western blot and dual-luciferase reporter gene analyses, we identified nuclear factor kappa B (NF-κB) repressing factor (NKRF) as the miR-494 target in cardiomyocytes. E_2_ was found to inhibit NKRF, thus activating NF-κB through a miR-494-dependent mechanism. In addition, the protective effects of E_2_ and miR-494 against oxidative stress in cardiomyocytes were eliminated by the NF-κB inhibitor. In summary, this study demonstrates for the first time that estrogen inhibits NKRF expression through ERα-mediated upregulation of miR-494 in cardiomyocytes, leading to the activation of NF-κB, which in turn results in an increase in antioxidative defense. ERα-mediated upregulation of miR-494 may contribute to estrogen protection of cardiomyocytes against oxidative stress.

## Introduction

Cardiac diseases are a growing public health problem (1). Intriguingly, observational studies provide strong support for significant gender differences in the incidence and prognosis of cardiac diseases (2). Premenopausal women face a lower incidence of myocardial dysfunction, ventricular hypertrophy, heart failure, and myocardial ischemia than age-matched men. This gender advantage is lost, however, once women become postmenopausal (3, 4). These findings have attracted great attention on the cardioprotective role of estrogens, which is supported by a number of clinical and animal studies (5–7).

Estrogens have been demonstrated to exert pleiotropic cardioprotective effects, among which antioxidative stress is one of the key effects linking estrogens to cardioprotection (8). Oxidative stress plays a pivotal role in the initiation and progression of cardiac diseases including myocardial ischemia/reperfusion (I/R) injury, ventricular hypertrophy, and heart failure. Estrogens have been reported to prevent oxidative stress-induced apoptosis in cardiomyocytes by counteracting mitochondrial reactive oxygen species generation (9). Prior studies including ours also demonstrate that estrogens may exert cardioprotection *via* increasing myocardial antioxidants and suppressing myocardial oxidative stress in animal models of estrogen deprivation (10, 11), chronic volume overload (12), and pressure overload-induced hypertrophy (13). Thus far, the mechanisms by which estrogens protect cardiomyocytes against oxidative stress remain largely unknown. We speculate that estrogen-regulated microRNAs (miRs) may be involved.

MicroRNAs are a class of endogenous small noncoding RNAs that negatively regulate the stability and translation of target protein-coding mRNAs at the 3′ untranslated region (UTR) (14). The dysregulation of miRs has been reported in a variety of cardiac diseases (15, 16). In particular, Queirós et al. reported that estrogen regulates a miRs network including miR-21, -24, -27a, -27b, -106a, and -106b in female cardiac fibroblasts, thereby modulating a spectrum of genes involved in cardiac fibrosis and remodeling (17). However, the estrogen-responsive miRs in cardiomyocytes still remain to be elucidated.

In the present study, we first used a miRs microarray screening approach to address miRs expression profiling in estrogen-treated cardiomyocytes. We discovered an increase in miR-494, which is known to exert cardioprotective effects against I/R-induced injury (18). We hypothesized that the upregulation of miR-494 might contribute to estrogen-mediated cardioprotection against oxidative stress.

## Materials and Methods

### Cell Culture

The preparation of the rat neonatal cardiomyocyte culture was as described previously (10, 11). Briefly, ventricle tissues were collected from rats that were up to 3 days old, and then minced in a dissociation buffer (in mmol/L: 116 NaCl, 20 HEPES, 0.8 Na_2_HPO_4_, 5.6 glucose, 5.4 KCl, 0.8 MgSO_4_, pH of 7.35) into 1 mm^3^ particles. Serial digestions were performed in a dissociation buffer containing 0.1% trypsin and 0.05% collagenase type II (Worthington Biochemical, Freehold, NJ, USA) at 37°C. Cell pellets were resuspended in DMEM containing 10% fetal bovine serum (FBS) and placed in culture dishes at 37°C for 1 h to allow for the selective attachment of nonmyocytes (primarily cardiac fibroblasts). The cardiomyocyte-enriched fraction (>95% cardiomyocytes as determined by immunocytochemistry staining) was then seeded onto a 12-well culture plate (Corning, Inc., Cambridge, MA, USA) at a density of 1 × 10^5^ cells/cm^2^ and cultured in DMEM containing 15 mmol/L HEPES, 10% FBS, 0.1 mmol/L bromodeoxyuridine (BrdU), and antibiotics (100 U/mL penicillin and 100 mg/mL streptomycin) for 48 h. The culture medium was then exchanged for serum-free DMEM containing the same additives with the exception of BrdU.

The H9c2 myocardial cell line was originally obtained from the American Type Culture Collection and kindly provided by the Shanghai Institute for Biological Sciences. The cells were cultured in a DMEM medium containing 10% FBS at 37°C in 5% CO_2_–95% air.

### miRNA Microarrays

Rat neonatal cardiomyocytes treated with 17β-estradiol (E_2_) for 24 h were used for small RNA extraction with the miRcute miRNA isolation kit (TianGen) according to the manufacturer’s protocol. The miRNAs were dissolved in DEPC treated water and the concentration was determined by Nanodrop2000c (Thermo). miRNA microarrays were performed as previously described (19). Briefly, RNA samples were isolated, size fractionated, and labeled with Cy3 or Cy5. The samples were hybridized to a dual-channel microarray using the μParaflo microfluidics chips of LC Sciences (Houston, TX, USA). This array contained probes for rat, mouse, and human miRNAs listed in Sanger miRBase Release 11.0. The reverse transcription, cRNA synthesis, labeling, and hybridization with Affymetrix GeneChip Rat Genome 230 2.0 Array were conducted following the standard Affymetrix protocol. The background-subtracted data were normalized by the LOWESS and quantile normalization methods. The log2-fold change values were calculated for each miRNA by comparing their expression between any two epididymal regions. Meanwhile, the statistical significance of the fold change (*P* value) was inspected through a Student’s *t*-test. A volcano plot was used to portray the difference in miRNA expression between the two epididymal regions, in which the *x*-axis indicated the log2-fold change value and the *y*-axis indicated the negative log10 *P* value. The raw datasets of microarray results have been submitted to the NCBI GEO database (Accession number GSE106501. miRNA microarray results are available in Table S1 in Supplementary Material).

### Real-Time RT-PCR

Total RNA from the heart tissue or cardiomyocytes was extracted by a TRIzol reagent (Invitrogen), and then 2 µg RNA was reverse transcribed to generate cDNA by using superscript reverse Transcriptase (Invitrogen) with a special stem-loop primer for miR-22 and oligodeoxythymidine for mRNAs. Quantitative real-time PCR was performed using a MiniOpticon real-time PCR detection system (Bio-Rad Laboratories). The primer sequences for nuclear factor kappa B (NF-κB) repressing factor (NKRF) were designed based on the cDNA sequences in GeneBank. The following primers were used: sense 5′- GTTCTGCCAAACACTGGACC-3′ and anti-sense 5′-CTGAGATAGGCTCCCGTATGCCC-3′. The reaction solution comprised 2.0 µl diluted cDNA, 0.2 µM/L of each paired primer, 200 µM/L deoxynucleotide triphosphates, 1 U Taq DNA polymerase (Qiagen, Beijing, China), and 1× PCR buffer. SYBRGreen (Roche Ltd, Basel, Switzerland) was used as the detection dye. The annealing temperature was set at 58–62°C and amplification was set at 40 cycles. The temperature range to detect the melting temperature of the PCR product was set from 60 to 95°C. To determine the relative quantification of gene expression, the comparative Ct (threshold cycle) method with arithmetic formulae (2^−ΔΔ^Ct) was used (20). The mRNA levels were normalized relative to the house-keeping gene β-actin.

### Ovariectomy and Hormone Replacement

Female C57BL/6 mice at 6–8 weeks of age (18–22 g) were obtained from Shanghai SLAC Laboratory Animal Co. (Shanghai, China) and housed at controlled room temperature with free access to food and water under a 12-h light/dark cycle. All animal protocols were approved by the Ethical Committee of Experimental Animals of Second Military Medical University. Bilateral OVX or a sham operation (Sham) was performed under anesthesia with sodium pentobarbital (60 mg/kg, ip). After 2 weeks, OVX mice were divided into four subgroups, and they were treated subcutaneously with the solvent sesame oil (30 μl/day, Sigma-Aldrich, St. Louis, MO, USA), E_2_ (30 μg/kg/day, Sigma-Aldrich), estrogen receptor ERα selective agonist PPT (30 μg/kg/day, Tocris bioscience, Bristol, UK) and ERβ selective agonist DPN (30 μg/kg/day, Tocris bioscience) for four weeks, as previously described (10, 11). All of the mice were then sacrificed for tissue collection. To avoid the influence of the estrus cycle, sham mice were sacrificed at the diestrus phase. Plasma E_2_ was determined with a commercial RIA kit (Sino-UK Institute of Biological Technology, Shanghai, China).

### 3-[4,5-Dimethylthiazol-2-yl]-2,5-Diphenyl Tetrazolium Bromide (MTT) Assay

Cell viability was evaluated by an MTT assay based on the reduction of MTT (Sigma-Aldrich) by functional mitochondria to formazan, as described previously (21).

### Lactate Dehydrogenase (LDH) Activity

Lactate dehydrogenase release into culture supernatants of the H9c2 myocardial cells was detected by colorimetric enzyme-linked immunosorbent assay, using the cytotoxicity detection kit (LDH) from Roche (Roche Diagnostics) as previously described (22).

### RNA Interference

The siRNAs for ERα were designed and synthesized by GenePharma Corporation (Shanghai, China). The target sequences for ERα was: 5′-CAGGTCCAATTCTGACAAT-3′. The negative control siRNA sequence was scrambled without any specific target: 5′-TTCTCCGAACGTGTCACGT-3′. The transfection of siRNA was performed by using a siPORT NeoFx transfection agent (Ambion, Austin, TX, USA) according to the manufacturer’s instructions.

### Dual-Luciferase Assay

The wild-type 3′UTR and the miR-494 “seed” mutant 3′UTR of NKRF were synthesized *in vitro* and cloned into the psi-CHECK2 luciferase reporter plasmid (Promega). The H9c2 cells were co-transfected with psiCHECK-2 plasmid containing wild-type or mutant derivatives, along with the miRNA control or miR-494 mimic. Lysates were collected 24 h after transfection and the luciferase activity was measured by a dual-luciferase reporter system (Promega).

### Western Blot Analysis

The proteins of the H9c2 cardiomyocytes were lysed with cold RIPA lysis buffer (Beyotime). The protein load was 30 µg/lane in 10% SDS-PAGE, and it was subsequently transferred to nitrocellulose membranes. The bolts were blocked with 5% skim milk powder in 0.1%tris-buffered saline/Tween20 for 2 h and incubated with antibodies (Santa Cruz, Cat. No. sc-365568) against NKRF overnight at 4°C at a dilution of 1:1,000. Then, the membrane was incubated with a secondary horseradish peroxidase-conjugated antibody for 1 h at room temperature. The immunoreactive proteins were visualized using the enhanced chemiluminescence western blotting detection system (Santa Cruz). The chemiluminescent signal from the membranes was quantified by a GeneGnome HR scanner using GeneTools software (SynGene). To control sampling errors, the ratio of band intensities to β-actin was obtained to quantify the relative protein expression level.

### Statistical Analysis

All data were expressed as the mean ± SEM. For illustrative purposes, some results are presented as the mean percent control ± SEM. When comparing multiple groups, one-way ANOVA was performed. When ANOVA showed significant statistical differences (*P* < 0.05), comparisons between each groups were conducted using the Student–Newman–Keuls test. SPSS 13.0 statistical software was used for the data analysis. *P* < 0.05 was considered statistically significant.

## Results

### E_2_ Stimulates miR-494 Expression *via* ERα in Both Cardiomyocytes and the Myocardium of Female Mice

**Figure 1:**
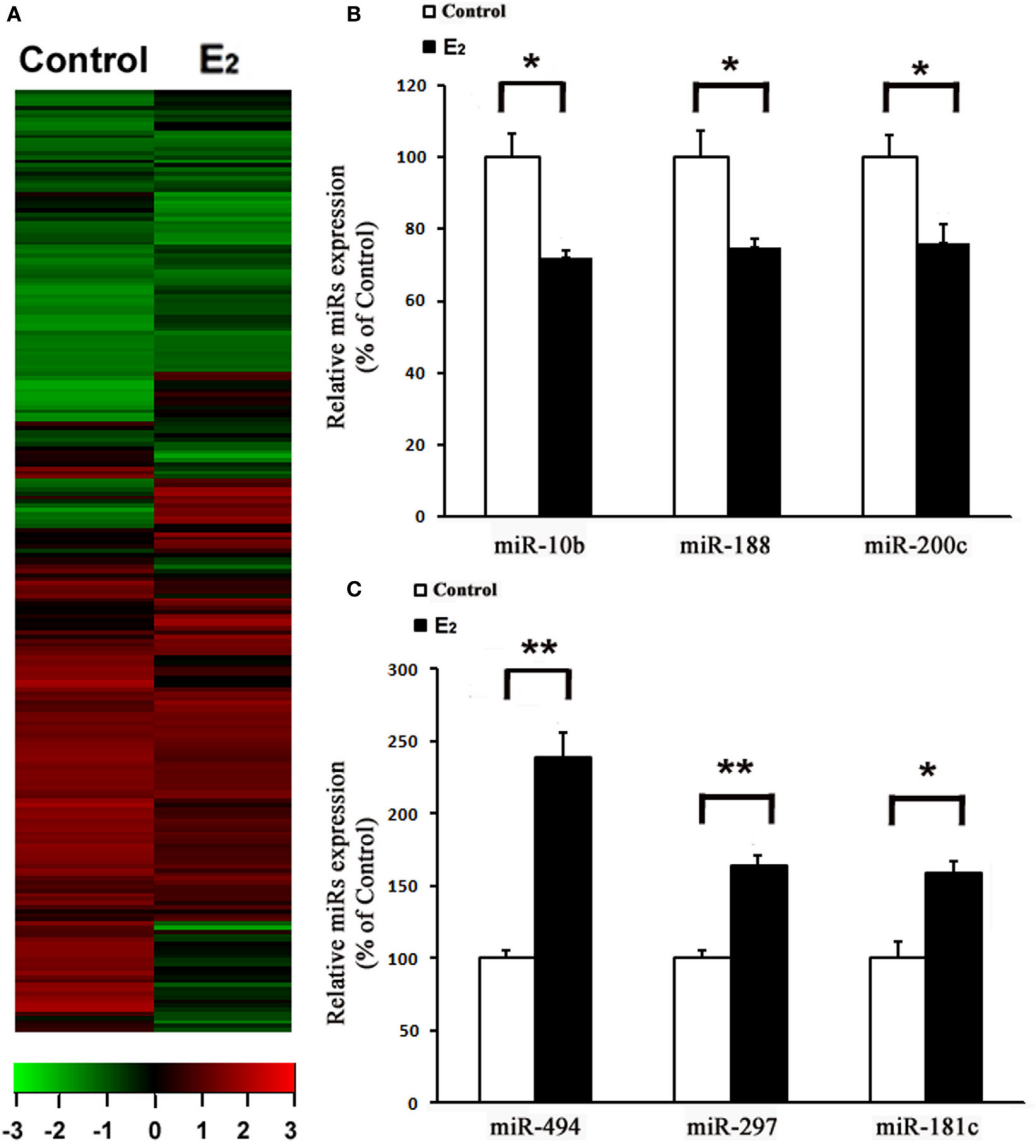
E_2_ regulates microRNAs (miRs) expression in cardiomyocytes. **(A)**, Heatmap image of microarray data, illustrating differentially expressed miRs between the control and E_2_-treated cardiomyocytes (*n* = 3). The scale is −3 to +3 in log10. Red indicates increased expression, and green indicates decreased expression. **(B,C)** Expressions of downregulated **(B)** and upregulated **(C)** miRs were determined by qRT-PCR by performing three independent experiments. All bar graphs represent the means ± SEM. *P < 0.05, **P < 0.01.

After comparing the miRNA expression profile between the control and E_2_-treated primary cultured rat cardiomyocytes using an array-based miRNA profiling (Figure 1A), six differentially expressed miRNAs were identified by qRT-PCR. Three were downregulated (miR-10b, miR-188, and miR-200c) and three were upregulated (miR-494, miR-297, and miR-181c) (Figures 1B,C). Because miR-494 is known to exert cardioprotective effects (18), we then further investigated the effect of E_2_ on miR-494 expression.

**Figure 2:**
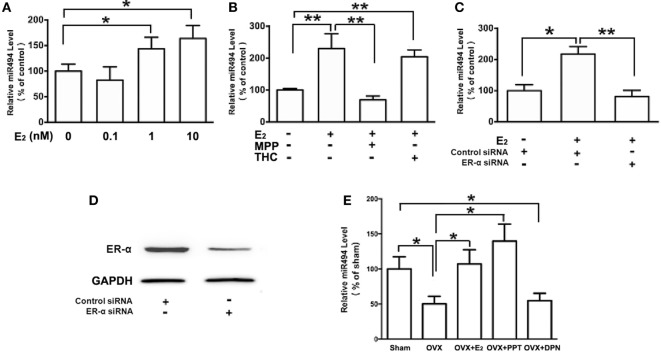
ERα mediates the regulation of miR-494 expression by E_2_ in cardiomyocytes and myocardium. **(A)** H9c2 cells were stimulated with E_2_ at indicated doses for 24 h. Quantitative real-time RT-PCR was used to determine miR-494 expression in cardiomyocytes by performing four independent experiments. **(B)** Cells were treated with E_2_ (10 nmol/L) in the absence or presence of ERα-specific antagonist MPP (1 µmol/L), or ERβ-specific antagonist THC (1 µmol/L) for 24 h. Quantitative real-time RT-PCR was used to determine miR-494 expression in cardiomyocytes by performing four independent experiments. **(C)** Cells were transfected with Control siRNA or ERα siRNA for 24 h and then treated with E_2_ (10 nmol/L) for another 24 h. Quantitative real-time RT-PCR was used to determine miR-494 expression in cardiomyocytes by performing four independent experiments. **(D)** Cells were transfected with Control siRNA or ERα siRNA for 24 h. Western blot analysis were used to determine ERα protein expression in cardiomyocytes by performing four independent experiments. **(E)** Bilateral ovariectomy was performed on female mice to deplete endogenous estrogens. Exogenous E_2_, ERα selective agonists (PPT) and ERβ selective agonists (DPN) were subcutaneously administered for four weeks. Quantitative real-time RT-PCR was used to determine miR-494 expression in the myocardium (*n* = 8 in each group). All bar graphs represent the mean ± SEM. *P < 0.05, **P < 0.01.

As shown in Figure 2A, the treatment of H9c2 with increasing concentrations of E_2_ (0.1–10 nM) caused significant increases in the miR-494 levels in a dose-dependent manner over a 24-h incubation period. The effect of E_2_ on miR-494 expression was completely eliminated by the ERα-selective antagonist MPP, but not by the ERβ-selective antagonist THC (Figure 2B). In addition, E_2_-induced miR-494 expression in cardiomyocytes was eliminated by the ERα siRNA treatment (Figure 2C). Transfection of ERα siRNA resulted in an approximately 80% decrease in ERα expression in the H9c2 cells (Figure 2D).

We then examined the roles of ERα and ERβ in the estrogen upregulation of miR-494 expression *in vivo*. To verify that the ovariectomy was successful, the weight of the uterus and level of E_2_ in the serum were determined. The level of serum E_2_ was found to be significantly lower in OVX mice than that in sham mice (data not shown). The weight of the uterus was also significantly decreased in the OVX group compared with the sham group (data not shown). As shown in Figure 2E, the ovariectomy resulted in decreased miR-494 expression in the myocardium of female mice. The E_2_ and ERα-specific agonist PPT treatment for 4 weeks significantly increased miR-494 expression in the myocardium whereas the ERβ-specific agonist DPN treatment did not affect miR-494 expression.

### Upregulation of miR-494 Expression Contributes to the Protective Effects of E_2_ Against Oxidative Stress in Cardiomyocytes

**Figure 3:**
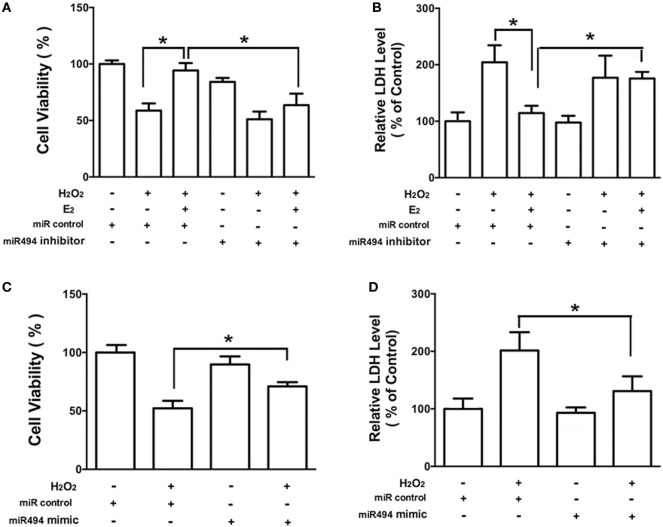
miR-494 contributes to the protective effects of E_2_ against oxidative stress in cardiomyocytes. **(A,B)** H9c2 cells were transfected with control microRNA (miR) or miR-494 inhibitor for 24 h and then treated with E_2_ (10 nmol/L) for another 24 h. Cells were then exposed to H_2_O_2_ (200 µmol/L) for 4 h. Overall cell viability was assessed by MTT **(A)** and supernatant lactate dehydrogenase (LDH) concentration **(B)**. **(C,D)** H9c2 cells were transfected with control miR or miR-494 mimics for 24 h. Cells were then exposed to H_2_O_2_ (200 µmol/L) for 4 h. Overall cell viability was assessed by MTT **(C)** and supernatant LDH concentration **(D)**. All bar graphs represent the means ± SEM from four independent experiments. *P < 0.05.

As shown in Figure 3, H_2_O_2_ (200 µmol/L) treatment of cardiomyocytes caused cell damage by showing an increase in the release of LDH as well as a decrease in cell viability. E_2_ at 10 nmol/L protected cardiomyocytes against the insult of H_2_O_2_ as evidenced by the increased cell survival and reduction of LDH release (Figures 3A,B). Moreover, the cardioprotective effects of E_2_ were significantly reduced in the presence of the inhibitor of miR-494 (Figures 3A,B). By contrast, the miR-494 mimic protected cardiomyocytes against the insult of H_2_O_2_ as evidenced by the increased cell survival and reduction of LDH release (Figures 3C,D).

### miR-494 Targets NF-κB Repressing Factor (NKRF), Thus Activating NF-κB

**Figure 4:**
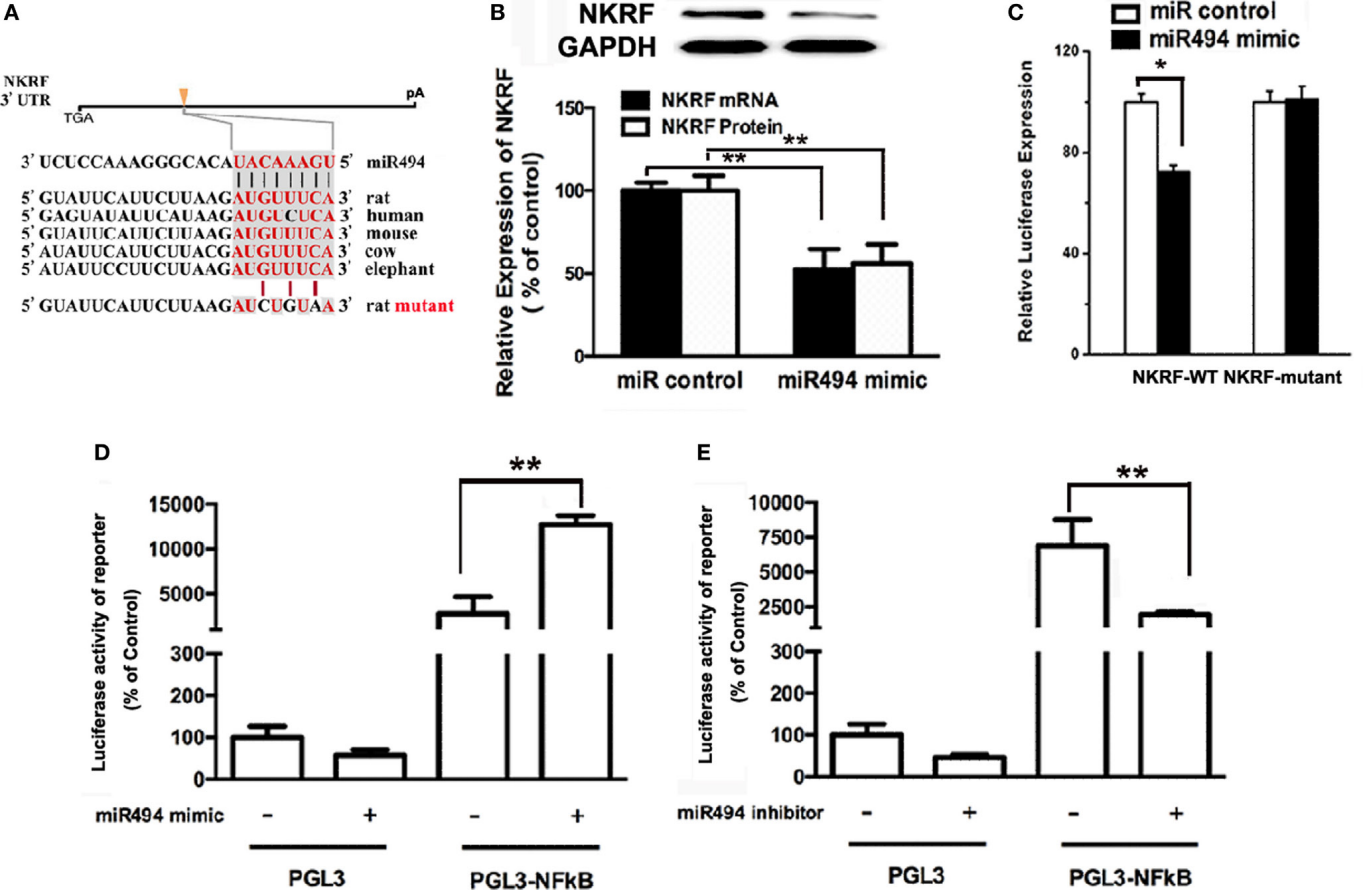
miR-494 targets NF-κB repressing factor (NKRF), thus activating NF-κB. **(A)** NKRF contained highly conserved miR-494-mRNA interaction motifs within their 3′ untranslated regions (UTRs), and the sequences of the wild-type (WT) and mutant 3′UTR of NKRF for the luciferase reporter assay are shown. **(B)** H9c2 were transfected with microRNA (miR) control or miR-494 mimics (200 nM) for 24 h. Quantitative real-time RT-PCR and western blot analysis were used to determine the NKRF mRNA and protein expression in cardiomyocytes, respectively. **(C)** H9c2 co-transfected with 0.4 µg of WT or mutant NKRF 3′UTR reporter plasmids in the presence of miR control or miR-494 mimics (200 nM). 24 h later, luciferase activity was measured using dual-luciferase assay. **(D)** H9c2 co-transfected with 0.4 µg of pGL3 or pGL3-NFκB reporter plasmids in the presence of miR control or miR-494 mimic (200 nM). 24 h later, luciferase activity was measured using dual-luciferase assay. **(E)** H9c2 co-transfected with 0.4ug pGL3 or pGL3-NFκB reporter plasmids in the presence of miR control or miR-494 inhibitor (200 nM). 24 h later, luciferase activity was measured using dual-luciferase assay. All bar graphs represent the mean ± SEM from four independent experiments. *P < 0.05, **P < 0.01.

MicroRNAs are known to negatively regulate the stability and translation of target protein-coding mRNAs at the 3′UTR (14). To identify potential targets for miR-494, we used a consensus approach with three widely used types of software (miRanda, TargetScan, and PicTar) to perform the target prediction. After overlapping prediction results, NKRF, a suppression factor for NF-κB, was selected to be a putative miR-494 target gene. The mRNA of NKRF contains a putative binding site for miR-494 in its 3′-UTR, and each site is broadly conservative among mammals (Figure 4A). As shown in Figure 4B, transfection with miR-494 significantly decreased both the mRNA and protein expression of NKRF in cardiomyocytes. To address whether NKRF was directly regulated by miR-494, we transfected NKRF 3′UTR luciferase reporter constructs together with a miR-494 mimic into cardiomyocytes and observed a significant reduction in luciferase activity in the co-transfection with miR-494 compared with that in the cells co-transfected with the nontargeting miR control (Figure 4C). By contrast, no reduction in luciferase activity was detected upon co-transfection of miR-494 when the putative miR-494 binding sequence in the NKRF 3′UTR luciferase reporter construct was mutated (Figure 4C). These findings suggest that NKRF expression is suppressed in cardiomyocytes by miR-494 binding to response elements in its 3′UTR.

It has been well-recognized that NKRF negatively regulates the activity of NF-κB through a direct protein–protein interaction (23). To determine the effect of miR-494 on NF-κB activity, H9c2 cells were transfected with an NF-κB report plasmid. As shown in Figures 4D,E, we found that the miR-494 mimic significantly stimulated, while the miR-494 inhibitor decreased the luciferase activity of the NF-κB report gene. Taken together, these findings suggest that miR-494 targets NKRF, thus activating NF-κB.

### E_2_ Inhibits NKRF, Thus Activating NF-κB Through a miR-494-Dependent Mechanism

**Figure 5:**
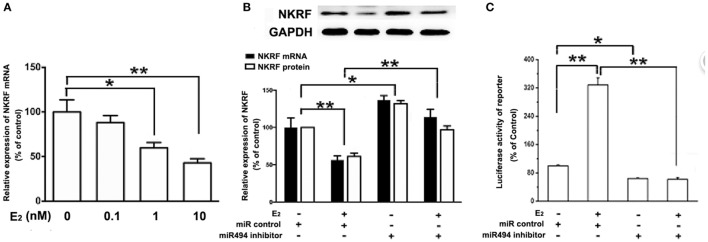
E_2_ inhibits (NF-κB) repressing factor (NKRF), thus activating NF-κB through a miR-494-dependent mechanism. **(A)** H9c2 cells were stimulated with E_2_ at indicated doses for 24 h. Quantitative real-time RT-PCR was used to determine NKRF mRNA expression in cardiomyocytes. **(B)** H9c2 cells were transfected with control microRNA (miR) or miR-494 inhibitor for 24 h and then treated with E_2_ (10 nmol/L) for another 24 h. Quantitative real-time RT-PCR and western blot analysis were used to determine NKRF mRNA and protein expression in cardiomyocytes, respectively. **(C)** H9c2 co-transfected with 0.4 µg of pGL3 or pGL3-NFκB reporter plasmids in the presence of miR control or miR-494 inhibitor (200 nM) for 24 h, and then treated with E_2_ (10 nmol/L) for another 24 h. Luciferase activity was measured using dual-luciferase assay. All bar graphs represent mean ± SEM from four independent experiments. *P < 0.05, **P < 0.01.

As shown in Figure 5A, the treatment of H9c2 with increasing concentration of E_2_ (0.1–10 nM) caused significant decreases in the NKRF levels in a dose-dependent manner over a 24-h incubation period. The treatment of the cells with E_2_ significantly increased the luciferase activity of the NF-κB report gene (Figure 5C). Notably, both the inhibitory effect of E_2_ on NKRF expression and the stimulatory effect of E_2_ on NF-κB activity were significantly reduced in the presence of the miR-494 inhibitor (Figures 5B,C).

### The Protective Effects of E_2_ and miR-494 Against Oxidative Stress in Cardiomyocytes Are Eliminated by the NF-κB Inhibitor

**Figure 6:**
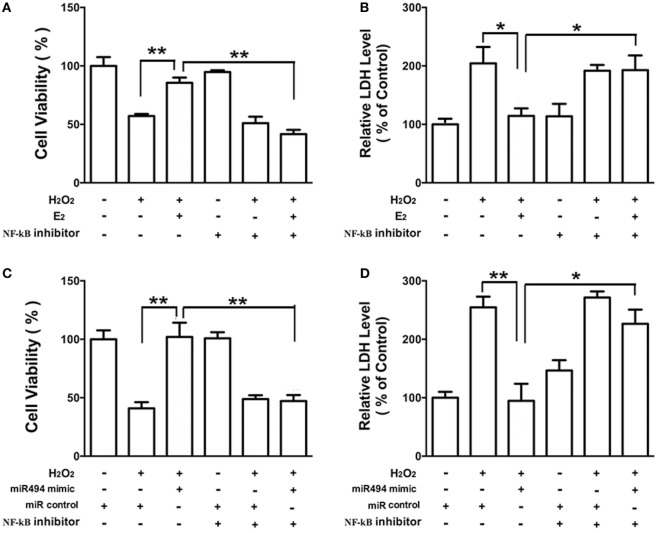
The protective effects of E_2_ and miR-494 against oxidative stress in cardiomyocytes are eliminated by NF-κB inhibitor. **(A,B)** H9c2 cells were incubated with E_2_ (10 nmol/L) in the absence or presence of NF-κB inhibitor (PDTC, 1 µmol/L) for 24 h. Cells were then exposed to H_2_O_2_ (200 µmol/L) for 4 h. Overall cell viability was assessed by MTT **(A)** and supernatant lactate dehydrogenase (LDH) concentration **(B)**. **(C,D)** H9c2 cells were transfected with control microRNA (miR) or mimics of miR-494 in the absence or presence of NF-κB inhibitor (PDTC, 1 µmol/L) for 24 h. Cells were then exposed to H_2_O_2_ (200 µmol/L) for 4 h. Overall cell viability was assessed by MTT **(C)** and supernatant LDH concentration **(D)**. All bar graphs represent the mean ± SEM from four independent experiments. *P < 0.05, **P < 0.01.

As shown in Figure 6, the H_2_O_2_ (200 µmol/L) treatment of cardiomyocytes caused cell damage by showing an increase in the release of LDH and a decrease in cell viability. Both E_2_ (Figures 6A,B) and the miR-494 mimic (Figures 6C,D) protected cardiomyocytes against the insult of H_2_O_2_ as evidenced by increased cell survival and the reduction in LDH release. These cardioprotective effects of E_2_ and the miR-494 mimic were significantly reduced in the presence of PDTC (1 µmol/L), an NF-κB inhibitor.

## Discussion

In this study, we tested the hypothesis that estrogenic regulation of miRNA expression is involved in estrogen-mediated cardioprotection against oxidative stress. miRNAs are powerful endogenous inhibitors of target genes at the posttranscriptional levels. Using array-based miRNA profiling, we found that six miRs were significantly regulated by estrogen treatment, with three upregulated and three downregulated in cardiomyocytes. Of these six miRs, miR-494 had the highest expression and the most significant difference. The cardioprotective effect of miR-494 against I/R-induced injury has been reported. However, the potential role of miR-494 in estrogen-mediated cardioprotection has not been explored.

Previous studies have described a link between miR-494 and cellular injuries in a cell-specific manner. For example, miR-494 contributes to inflammatory or adhesion molecule-induced kidney injury after I/R by inhibiting the expression of activating transcription factor 3 (24). miR-494 inhibition protects nucleus pulposus cells from TNF-α-induced apoptosis by targeting JunD (10, 11). By contrast, Sun et al. reported that miR-494 upregulates HIF-1α expression through activating the PI3K/Akt pathway, thus protecting against hypoxia-induced apoptosis in human liver cell line L02 cells (25). MicroRNA-494 has also been found to improve functional recovery and inhibits apoptosis in rats after spinal cord injury (26). In the myocardium, Wang et al. demonstrated that miR-494 has cardioprotective effects against ischemia/reperfusion-induced cardiac injury *via* targeting apoptotic factors (18). Consistent with the findings of Wang et al., this study indicated that miR-494 *per se* exerted protective effects against oxidative stress-induced injuries in cardiomyocytes. In addition, the protective effects of estrogen against oxidative stress were blocked by the miR-494 inhibitor, suggesting that miR-494 contributed to estrogen-mediated cardioprotection.

The estrogenic modulation of miRNA expression has been widely described, especially in the pathogenesis of tumor development and metastasis (14). As for miR-494, previous studies show that estrogen treatment results in the upregulation of miR-494 expression in the human mammary adenocarcinoma cell MCF-7 (27) and the human hepatocarcinoma cell line HuH-7 (28), thus indicating a possible role of miR-494 in the estrogen-mediated downregulation of tissue factor pathway inhibitor α and protein S, respectively. In the present study, a dose-dependent induction of miR-494 expression levels was detected in cardiomyocytes treated with estrogen. In addition, the inhibitory effect of estrogen on NKRF, a target gene of miR-494, was blocked by the miR-494 inhibitor. These findings suggest a possible role for miR-494 in the estrogenic regulation of NKRF at the posttranscriptional level.

Estrogens exert their biological effects *via* numerous mechanisms, one of which is through the two classic nuclear estrogen receptors, ERα and ERβ. Although both are involved in estrogen cardioprotection, the relative contribution and prevalence of each receptor may be dependent on the type of cardiac injury. ERα has been found to exert the cardioprotective effect of estrogens against acute I/R injury through upregulating some endogenous cardioprotective molecules such as stromal cell-derived factor-1 (29). ERα even supports the survival of cardiomyocytes indirectly through post-infarct cardiac c-kit+ cells accumulating in the peri-infarct myocardium (30). Our previous study also demonstrated that ERα mediates estrogen upregulation of CSE in cardiomyocytes, which contributes to the protective effects of estrogen against oxidative stress (10, 11). For the role of ERβ, a number of studies have demonstrated that ERβ exhibits myocardial protection following ischemia (31–33). Recently, the role for ERβ in reducing hypertrophy in females was also reported (34). However, the present study showed that ERα but not ERβ mediated E_2_ maintenance of miR-494 expression in cardiomyocytes and the myocardium, again corroborating the distinct functions of ERα and ERβ in the heart.

*In silico* analysis of the NKRF 3′-UTR sequence identified one putative miR-494 binding site. In our study, we performed *in vitro* validation of miR-494 to negatively regulate NKRF expression in cardiomyocytes. The direct interaction of miR-494 with NKRF mRNA was assessed by NKRF 3′-UTR-dependent luciferase activity and co-transfection with a miR-494 mimic that led to significantly suppressed luciferase activity. Mutation of the predicted miR-494 binding site abrogated the effect of miR-494 on the NKRF 3′-UTR luciferase activity. Overexpression of the miR-494 mimic also led to significantly reduced NKRF mRNA and protein levels that was consist with the luciferase data. NKRF is well-recognized as a suppression factor for NF-κB (23). We found that the miR-494 mimic significantly stimulated, while the miR-494 inhibitor decreased the luciferase activity of the NF-κB report gene, suggesting that miR-494 targets NKRF, thus activating NF-κB. To our knowledge, this is the first report indicating that miR-494 directly regulates NKRF expression in cardiomyocytes and supports the potential involvement of miR-494 in the regulation of the NKRF/NFκB signaling pathway.

The transcription factor NF-κB has been implicated in the regulation of immune cell maturation, cell survival, and inflammation in many cell types, including cardiac myocytes. Prolonged activation of NF-κB appears to be detrimental, promoting heart failure by eliciting signals that trigger chronic inflammation through enhanced elaboration of cytokines, leading to endoplasmic reticulum stress responses and cell death (35). However, accumulating studies have documented a potent role for NF-κB in the regulation of cardiac myocyte survival through the repression of apoptotic cell death triggered by hypoxia, chemotherapeutic agent, or ischemic/reperfusion myocardial injury (36–38).

Oxidative stress is considered one of the main causative factors in various cardiovascular disorders (39). Given that NF-κB targets a number of important cellular antioxidants including manganese superoxide dismutase (MnSOD, or SOD2), Copper-Zinc superoxide dismutase (Cu,Zn-SOD, or SOD1), ferritin heavy chain, metallothionein-3 (MT3), and NAD(P)H dehydrogenase [quinone] 1 (NQO1), the protective effects of NF-κB against oxidative stress-induced injuries have been widely reported in a variety of cell types including cardiomyocytes (39, 40). For example, the NF-κB signaling pathway attenuates oxidative stress and plays a protective role in cardiomyocytes through the regulation of MnSOD in a setting of acute pressure overload (41). In addition, some agents, such as hesperetin (38), SIRT3 (42), and metformin (43), have shown beneficial effects against oxidative stress-induced cardiotoxicity, which is, at least partly, mediated by the activation of NF-κB. In the myocardium, estrogens have been shown to exert protective effects against oxidative stress. Estrogen is also found to activate NF-κB in adult cardiomyocytes (44). Consistent with these studies, we found that estrogen activated NF-κB through a miR-494-dependent mechanism. The protective effects of both miR-494 and estrogen against oxidative stress in cardiomyocytes were eliminated by the NF-κB inhibitor. Taken together, these results suggest that miR-494 may link estrogenic cardioprotection with NF-κB activation in cardiomyocytes.

Although numerous experimental *in vitro* and *in vivo* studies have provided reliable evidence supporting estrogen’s cardiovascular benefits (5, 8, 9, 12, 30), clinical trials report contradictory results regarding cardiovascular effects of menopausal hormone use (45). For example, the Women’s Health Initiative (WHI) clinical trial enrolls participants whose average age at initiation of treatment was 63 years with the age range of 50–79 years (46). Women with a uterus were given oral conjugated equine estrogen (CEE) with continuous combined medroxyprogesterone acetate (MPA). Women who had undergone hysterectomy alone or with either unilateral or bilateral oophorectomy were treated with CEE alone. The CEE + MPA study of the WHI demonstrated increased adverse cardiovascular events including cardiac events, strokes, and pulmonary embolisms. However, in the CEE alone cohort, the combined endpoints of coronary heart disease events and coronary revascularization were lower in women randomized to treatment who were between the ages of 50–59 compared to women in age strata > 60 years (47). In an 11-year cumulative follow-up study of the CEE alone cohort, myocardial infarction was lower in the 50–59 age strata than in women who had been randomized to treatment at older ages (48). Additionally, a large Danish open-label study (Danish Osteoporosis Prevention Study) enrolled women between the ages of 45–58 years (49). The treatments were either triphasic estradiol and norethisterone acetate or estradiol a day if they had a hysterectomy. At 11 years follow-up, there were significantly fewer myocardial infarctions and lower incidence of heart failure in the treated women compared to the control group. These clinical trials suggest that the timing of initiation of hormone therapy in relation to menopause onset or age might influence cardiovascular risk. The limitation of the present study is that it seems difficult to extrapolate the data obtained from experimental studies to humans to indicate estrogen’s cardiovascular benefits.

In summary, this study demonstrates for the first time that E_2_ stimulates miR-494 expression *via* ERα in both cardiomyocytes and the myocardium of female mice. ERα-mediated upregulation of miR-494 may contribute to estrogen protection of cardiomyocytes against oxidative stress. Furthermore, we identify NKRF as a miR-494 target in cardiomyocytes. Estrogen inhibits NKRF expression through ERα-mediated upregulation of miR-494 in cardiomyocytes, leading to the activation of NF-κB, which in turn results in an increase in antioxidative defense.

## Ethics Statement

All animal protocols were approved by the Ethical Committee of Experimental Animals of Second Military Medical University.

## Author Contributions

X-YZ and XN drafted the manuscript; Z-PT, WZ, and J-kD prepared figures; X-YZ and J-QL edited and revised the manuscript critically for important intellectual content; Z-PT, WZ, J-kD, XN, X-YZ, and J-QL approved the final version of the manuscript submitted.

## Conflict of Interest Statement

The authors declare that the research was conducted in the absence of any commercial or financial relationships that could be construed as a potential conflict of interest.
